# Protective effect of intermittent clamping of the portal triad in the rat liver on liver ischemia-reperfusion injury

**Published:** 2011-06-01

**Authors:** Krzysztof Helewski, Grazyna Kowalczyk-Ziomek, Eugeniusz Czecior, Grzegorz Wyrobiec, Marzena Harabin-Slowinska, Malgorzata Juszko-Piekut, Bogumila Braczkowska, Jadwiga Josko

**Affiliations:** 1 Department of Histology and Embryology, Medical University of Silesia, Zabrze, Poland; 2 Department of ENT, Medical University of Silesia, Zabrze, Poland; 3 Department of Hygiene, Bioanalysis and Environmental Studies, School of Pharmacy, Medical University of Silesia, Zabrze, Poland; 4 Department of Epidemiology, Medical University of Silesia, Katowice, Poland; 5 Department of Environmental Medicine and Epidemiology, Medical University of Silesia, Zabrze, Poland

**Keywords:** Ischemic preconditioning, Acute liver failure, Reperfusion injury, TNF-alpha, Apoptosis

## Abstract

**Background:**

Intermittent clamping (IC) of the portal triad is an effective method of protecting the liver from ischemia-reperfusion injury (IR). In clinical practice, this method is employed during a resection, but its mechanism is still not clear.

**Objectives:**

To evaluate the effect of IC on rat liver and determine its mechanisms.

**Materials and Methods:**

Wistar rats were submitted to 60-min IC (cycles of 12-min clamping followed by 4-min reperfusion), and the samples were collected after 1, 6, and 72 hrs of reperfusion. We determined the serum activity of alanine aminotransferase (ALT), and measured the concentration of TNF-α, malondialdehyde (MDA) and myeloperoxidase (MPO) in liver homogenates. The apoptosis of hepatocytes was evaluated immunohistochemically.

**Results:**

When compared to the IR rats, the activity of ALT decreased in the IC group in all periods of observation (the highest decrease of ~48% after 1 hr of reperfusion). When compared to the IR group, a statistically significant decrease (p < 0.05) in the TNF-α concentration (~33%) in the IC rats occurred only after 1 hr of reperfusion, and it was accompanied by a decrease in the MPO concentration after 1 and 6 hrs of reperfusion. IC reduces the effects of reactive oxygen species (ROS) activity, which has been confirmed by a statistically significant decrease in MDA concentration by 25%-35% in all studied periods. The limitation of hepatocytes apoptosis due to IC occurs in the early (~26%; p < 0.05) and late (~45%; p < 0.01) phases of reperfusion.

**Conclusions:**

The use of IC in early phase of reperfusion brings about a decrease in TNF-α release, which can be related to liver injury due to neutrophil infiltration and apoptotic cell reduction. It seems that the reduction of lipid peroxidation may also limit the liver injury.

## 1. Background

Liver ischemia-reperfusion (IR) injury is a serious clinical problem that occurs during resection surgery, organ transplantation and hemorrhagic shock [1][2][3][4]. Presently, two surgical procedures are employed to reduce the liver damage resulting from IR, namely, ischemic preconditioning and intermittent clamping (IC). The first attempt to limit the liver injury during warm ischemia was described in patients in 1987. The attempt involved interrupting a longer period of ischemia by intermittent clamping [5]. The mechanism utilized by IC to protect the liver from the effects of ischemia is still only partly known. However, it was shown that IC limits apoptosis of hepatocytes and endothelial cells of liver sinusoids [6][7][8]. It has been suggested that IC can reduce the number of apoptotic cells either by limiting the production of reactive oxygen species (ROS), or inhibiting the activation of Browicz-Kupffer cells (KC), thereby reducing the release of TNF-α-an important mediator of the liver ischemia-reperfusion injury [8][9]. The first of these alternatives has been supported by in vitro studies, which have proved that intermittent oxygen deprivation of hepatocytes reduces the production of ROS and hepatocytes damage, when compared to the total lack of oxygen[10]. The observations have been confirmed by animal studies, since, as compared to continuous inflow clamping, intermittent clamping (IC) significantly reduces oxidative stress and the organ injury [7][11][12].

## 2. Objectives

The objective of our study was to investigate the effect of IC of the portal triad in a rat liver on liver ischemia-reperfusion injury, and determine its mechanisms. To accomplish our objective, we evaluated hepatocytes injury on the basis of the serum activities of alanine aminotransferase (ALT). The damage resulting from ROS was evaluated by measuring the concentration of malondialdehyde (MDA) in liver homogenates. We also evaluated hepatocytes apoptosis and neutrophil infiltration, which indicated inflammation, by measuring myeloperoxidase (MPO) and TNF-α concentration.

## 3. Materials And Methods

Experiments were conducted under the consents of the local Ethics Committee for Animals Research (LECAR protocol No. 15/05) of the Medical University of Silesia. The study was performed on male Wistar rats weighing between 200 and 250 g. During the experiment, all rats were kept in standard breeding conditions (temperature of 21-23 °C, relative humidity of 65%-70%, and day/night cycle of 12/12 h). The animals were fed with standard Murigran feed and water ad libitum. The animals were divided into experimental groups containing six rats each:

1-Controls (rats without medical intervention) (K group)

2-Controls subjected to laparotomy examined after 1, 6, or 72 hrs (SO1, SO6 and SO72 groups, respectively).

3-Rats subjected to 60-min partial hepatic ischemia and 1, 6, or 72 hrs reperfusion (IR1, IR6 and IR72 groups, respectively).

4-Rats subjected to 60-min intermittent portal triad clamping (4 cycles of ischemia for 12 min followed by 3 reperfusions 4 min long) and 1, 6, or 72 hrs reperfusion (IC1, IC6 and IC72 groups, respectively).

All surgical procedures were conducted under general anesthesia after intra-peritoneal injection of ketamine (10 mg/kg body weight) and droperidol (0.25 mg/kg body weight). The rats in IR groups were subjected to partial hepatic ischemia (70%) followed by reperfusion, as described by Asakawa et al. [13]. The access to the liver was gained through a midline incision. Then ischemia was induced by occluding for 60 min the hepatic artery, hepatic vein and bile ducts to the left and median lobes with an atraumatic vascular clamp (Aesculap). Such a choice of ischemia and reperfusion model allows to prevent passive hyperemia of abdominal organs. Reperfusion was caused by removing the vascular clamps. In IR rats, the surgical field was covered with 0.9% NaCl dampened gauze swab; the gauze swab was left until the sample collection. In all groups, the interguments were stitched with double-layer 4-0 silicon thread. Then, the rats were again anesthetised, laparotomy was made in the previous incision site, and the left and median lobes (after IR) and cardiac blood samples were collected for further studies. In IC rats, the hepatic artery and vein and the bile duct were clamped for 12 min, and then unclamped for 3 min. The cycle was repeated in such a way to obtain 4 periods of 12-min ischemia which were interrupted by three periods of 4-min reperfusion within 60 min. Further proceedings were similar to those mentioned above. After centrifugation, plasma and serum samples obtained from the whole blood were portioned and frozen at 85 oC until analyzed. Liver samples were homogenised in a volume of phosphorous buffer (50 mmol/L, pH 6), five times greater than the tissue volume; they were then portioned and frozen at 85 oC. The samples for immunohistochemical evaluation were always collected from the left lobe. Liver samples were first fixed in buffered formalin and then embedded in paraffin. Then, sample slices 4-μm thick were placed on slides for immunohistochemical staining.

Serum ALT activity was determined by the method of Wroblewski-LaDue modified by Henry and Bergmeyer using the LDH-NADH complex [14][15][16]. The spectrophotometry was conducted at wavelength of 340 nm by RA-XT analyser (Technicon Instruments Corporation, Tarrytown, New York, USA). Concentrations of TNF-α and MPO in the liver homogenates were measured by a enzyme-linked immunosorbent assay (ELISA) using commercially available kits (R and D Systems, MN, USA; and HBT, Uden, Netherlands, respectively) according to the manufacturers' instructions. The measurements were conducted with ELx800 Microplate Reader and ELP-40 Microplate Strip Washer (BIO-TEK Instruments Inc., Winooski, VT, USA). The MDA concentration in the liver homogenates was measured by the Ohkawa method, using the reaction with tiobarbiturate acid. End-products of oxidized lipids degradation were expressed as the values of MDA concentrations [17]. The method was modified by adding butylated hydroxytoluene (BHT) and sodium sulfate to eliminate other compounds which could react with tiobarbiturate acid. Extinction was assayed using Shimadzu A160 spectrofluorometer (Shimadzu, Japan) at wavelengths of 515 and 552 nm (absorbance and emission, respectively). The assay of cells undergoing apoptosis was conducted immunohistochemically with M30 CytoDEATH kit (Roche Applied Sciences, Germany), using a 3-stage method (ABC). Apoptosis was evaluated under a light microscope in 30 visual fields, and the results were expressed as the percentage of hepatocytes that underwent apoptosis in relation to a total number of calculated hepatocytes. The obtained results were analysed statistically and expressed as mean ± SD. The Mann-Whitney U test for was used to compare the two groups. A p < 0.05 was considered statistically significant.

## 4. Results

**Figure 1:**
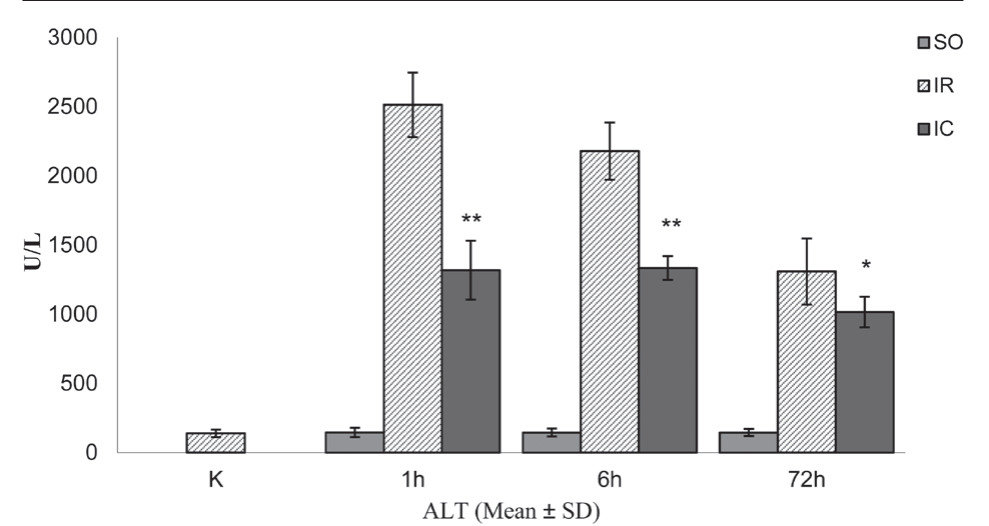
The mean serum activity of alanine aminotransferase (ALT) in the control and experimental rats subjected to 60-min continuous and intermittent ischemia and 1, 6, or 72 hrs of reperfusion. Error bars represent SD (n = 6).

The mean ± SD serum ALT activity of the controls without medical intervention (K group) was 140 ± 27 U/L (Figure 1). In the sham-operated rats, after one hour of reperfusion (SO1), the activity of ALT was similar to the value obtained for the K group. In the IR1 group, there was a marked increase in mean ± SD ALT activity (~1700%), to 2513 ±233 U/L, (p < 0.01 vs. K group). In the IC1 group, the mean ± SD ALT activity decreased to 1319 ± 212 U/L, 48% lower than IR1 (p < 0.01). After six hours of reperfusion, the ALT activity in the SO6 group (mean ± SD: 145 ± 29 U/L) was as low as in the SO1 group (Figure 1). In the IR6 group, the mean ± SD ALT activity (2179 ± 206 U/L) was significantly higher than that in the K and SO6 groups (~1460% and 1410%, respectively) (p < 0.01). In the IC6 group, the mean ± SD ALT activity decreased to 1335 ± 86 U/L, 29% lower than IR6 (p < 0.01). After 72 hrs of reperfusion, the ALT activity in SO72 was as low as in the K, SO1, SO6 groups (Figure 1). In the IR72 group, the mean ± SD ALT activity (1309 ± 239 U/L) was significantly higher than that in the C and SO72 groups (~839% and 805%, respectively) (p < 0.01). In the IC72 group, the mean ± SD ALT activity decreased to 1016 ± 111 U/L, 22% lower than IR72 group (p < 0.05).

**Figure 2:**
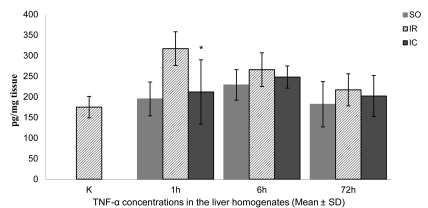
The mean TNF-α concentrations in the liver homogenates of the control and experimental rats subjected to 60-min continuous and intermittent ischemia and 1, 6, or 72 hrs of reperfusion. Error bars represent SD (n = 6).

The mean ± SD TNF-α concentration in the liver homogenates in the controls (K groups) was 175 ± 26 pg/mg (Figure 2). The mean ± SD concentration of TNF-α was slightly higher in sham-operated rats (SO1 group), at 195 ± 41 pg/mg tissue (Figure 2). In the IR1 group, the mean ± SD TNF-α concentration of 317 ± 41 pg/mg was ~80% higher than that in the controls (p < 0.01). The mean ± SD TNF-α concentration in the liver homogenates in the IC1 group decreased to 212 ± 78 pg/mg tissue (~33%) (p < 0.05). After six hours of reperfusion, the liver mean ± SD TNF-α concentration of 229 ± 37 μmol/g in the SO6 group was higher than that in the K and SO1 groups (Figure 2). In the IR6 group, the mean ± SD TNF-α concentration of 266 ± 41 pg/mg tissue was higher than that in the K and SO6 groups (~52% and 16%, respectively), but only the first difference was statistically significant (p < 0.01). Liver mean ± SD TNF-α was slightly but not significantly lower (248 ± 27 pg/mg tissue) in the IC6 compared to the IR6 group. After 72 hrs of reperfusion, the liver mean ± SD TNF-α concentration of 182 ± 55 pg/mg was similar to the values obtained for the K and SO1 groups, but it was lower than that in the SO6 group (Figure 2). The mean ± SD concentration of 217 ± 39 pg/mg tissue was higher than that in the K and SO72 group (~24% and 19%, respectively), but only the first difference was statistically significant (p < 0.05). In the IC72 group, the mean ± SD TNF-α concentration decreased to 191 ± 50 pg/mg tissue, which was lower than that in the IR72 (not significantly different).

**Figure 3:**
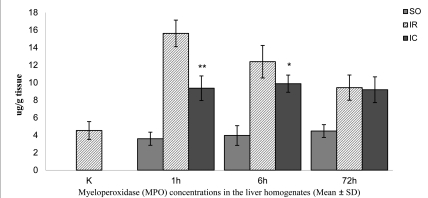
The mean myeloperoxidase (MPO) concentrations in the liver homogenates of the control and experimental rats subjected to 60-min continuous and intermittent ischemia and 1, 6, or 72 hrs of reperfusion. Error bars represent SD (n = 6).

The mean ± SD MPO concentrations in the liver homogenates in the K and SO1 groups were similar (4.53 ± 1.02 and 3.59 ± 0.75 μg/g tissue, respectively) (Figure 3). In IR1 liver homogenates, the mean ± SD MPO concentration was 15.62 ± 1.52 μg/g, a 250% increase compared to the K group (p < 0.01). The mean ± SD MPO concentration decreased by ~40% (9.26 ± 1.5 μg/g tissue) when compared to IR1 (p < 0.01). After six hours of reperfusion, the mean ± SD MPO concentration of 3.96 ± 1.13 μg/g tissue in the SO6 group was similar to the values obtained for the K and SO1 groups (Figure 3). In the IR6 group, the mean ± SD MPO concentration increased to 12.39 ± 1.86 μg/g tissue, and was higher than that in the K and SO6 groups (~174% and 213%, respectively) (p < 0.01). In the IC6, there was ~20% decrease in the MPO concentration compared to IR72 (p < 0.05). After 72 hours of reperfusion, the mean ± SD MPO concentration of 4.47 ± 0.73 μg/g tissue in SO72 was similar to the values obtained for the K, SO1 and SO6 groups (Figure 3). In the IR72 group the mean ± SD concentration of MPO increased to 9.43 ± 1.44 μg/g tissue which was higher than that in the K and SO72 groups (~108% and 111%, respectively) (p < 0.01). In the IC72 group, the mean ± SD MPO concentration decreased to 9.19 ± 1.47 μg/g tissue, ~3% lower than that in the IR72 group, which was statistically significant.

**Figure 4:**
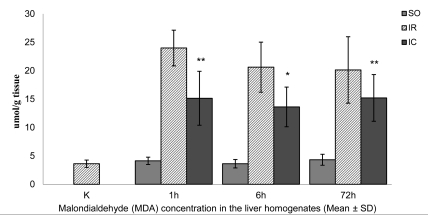
The mean malondialdehyde (MDA) concentration in the liver homogenates of the control and experimental rats subjected to 60-min continuous and intermittent ischemia and 1, 6, or 72 hrs of reperfusion. Error bars represent SD (n = 6).

**Figure 5:**
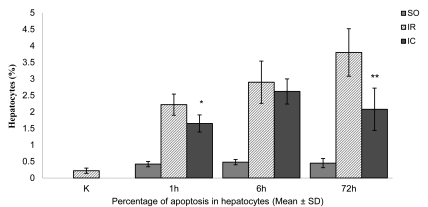
The mean percentage of apoptosis in hepatocytes of the control and experimental rats subjected to 60-min continuous and intermittent ischemia and 1, 6, or 72 hrs of reperfusion. Error bars represent SD (n = 6).

The mean ± SD MDA concentrations in the liver homogenates of the controls (K) and sham-operated (SO1) rats were similar, at 3.62 ± 0.65 and 4.13±0.59 μmol/g tissue, respectively (Figure 4). In IR1 rats, the mean ± SD MDA concentration was 23.99 ± 3.14 μmol/g tissue-an increase of 560% compared to the K group (p < 0.01). In the IC1 group, the mean ± SD MDA level decreased to 15.14 ± 4.75 μmol/g tissue, which was 37% lower than that in the IR1 group (p < 0.01). After six hours of reperfusion, the mean ± SD MDA concentration in SO6 rats decreased to as low as 3.62 ± 0.65 μmol/g tissue in the IR1 and SO1 groups (Figure 4). The mean ± SD MDA concentration in the IR6 group increased to 20.62 ± 4.41 μmol/g tissue, and was higher than that in the K and SO6 groups (~470% and 469%, respectively) (p < 0.01). In the IC6 group, the MDA concentration decreased by ~35% when compared to IR6 (p < 0.05). After 72 hours of reperfusion, the mean ± SD MDA concentration of 4.32 ± 0.97 μmol/g tissue in the SO72 group was as low as that in the K, SO1 and SO6 groups (Figure 4). The mean ± SD MDA concentration in the IR72 group increased to 20.13 ± 5.85 μmol/g tissue, and was higher than in the K and SO6 groups (~456% and 367%, respectively) (p < 0.01). In the IC72 group, the MDA concentration decreased by ~25% when compared to IR72 group, but the difference was not statistically significant. The mean ± SD proportion of hepatocytes that died of apoptosis in immunohistochemical preparations in the K group was 0.22% ± 0.08% (Figure 5). The proportion (0.42% ± 0.08%) was higher in the SO1 than in the K group (p < 0.01). The mean ± SD hepatocyte proportion of 2.22% ± 0.32% in IR1 group was higher than that in the C and SO1 groups (~909% and 429%, respectively) (p < 0.01). In the IC1 group, the mean ± SD hepatocytes proportion decreased to 1.65% ± 0.26%, which was ~26% lower than IR1 group (p < 0.05). After six hours of reperfusion, the proportion of hepatocytes that died of apoptosis in immunohistochemical preparations in the SO6 group (0.48% ± 0.08%) was markedly higher than that in the K group, and only slightly higher than that in the SO1 group (Figure 5). The mean ± SD hepatocyte proportion of 2.9% ± 0.64% in IR6 group was higher than that in the K and SO6 groups (~1218% and 504%, respectively) (p < 0.01). In the IC6 group, the mean ± SD hepatocytes proportion decreased to 2.62% ± 0.38% which was ~10% lower than IR6 group, but it was not statistically significant. After 72 hours of reperfusion, the proportion of hepatocytes that died of apoptosis in immunohistochemical preparations in the SO72 group (0.45% ± 0.14%) was markedly higher than that in the K group, and similar to the values obtained in the SO1 and SO6 groups (Figure 5). The mean ± SD hepatocyte proportion of 3.8% ± 0.72% in IR72 group was higher than that in the K and SO72 groups (~1627% and 744%, respectively) (p < 0.01). In the IC72 group, the mean ± SD hepatpcytes proportion decreased to 2.08% ± 0.64% which was ~45% lower than IR72 group (p < 0.01).

## 5. Discussion

Although it is not clear how this protective effect occurs, good results of IC of the portal triad have led to a common use of this method in practical medicine. However, the employment of IC during liver resection has a serious disadvantage, since every unclamping causes reperfusion and contributes to blood loss [18]. Nevertheless, the benefits of IC have been confirmed by clinical trials in patients after extensive liver resection (without cirrhosis), when the effects of both, ischemic preconditioning and IC were compared. It has been shown that the liver protection from injury is equally effective in case of both methods [19]. Literature described long periods of ischemia which were possible thanks to IC. The method has been proved to enable proper liver functioning in spite of 5-hr ischemia [20]. Nonetheless, the studies of different ischemic periods and reperfusion have not brought unequivocal results [21][22][23][24]. The determination of serum ALT activities is one of the most common assays of liver injury. Since ALT is only present in hepatocyte cytosol, the serum ALT activity indicates that cytoplasmic membranes of hepatocytes have been damaged, which can lead to their death. In our study, the protective effect of IC was strongest (according to ALT activity) after one and six hours of reperfusion. A smaller difference in obtained values between the IR72 and IC72 groups may suggest a weaker effect of the used method on mechanisms causing hepatocyte damage in further reperfusions. The ALT concentration in the IC rats in early (2 hrs) and late (24 hrs) phases of reperfusion in the experiment of Kimura et al. were in keeping with our results [25]. Similar results of the ALT activity in early phase of reperfusion (120 hrs) were obtained by van Wagensveld et al. in pigs subjected to 90-min intermittent portal triad clamping [26]. Whereas, after six hours of reperfusion, van Wagensveld et al. did not record any differences in the concentration of ALT between the pigs after continuous and intermittent 120-min clamping [27], their results were contradictory to our results. Such a difference may be attributed to longer ischemia, which can cause greater liver damage. During the first phase of liver injury due to IR, Kupffer cells are activated and become the main source of free oxygen radicals, as well as numerous mediators, including TNF-α. Not only can this cytokine cause apoptosis and attract neutrophils to the inflammation site, but also it is a direct stimulus of the formation of ROS and their metabolites. TNF-α (excluding IL-1) is one of the most important mediators produced by macrophages, the mediators which play key roles in the early stage of inflammation and activate endothelial cells and neutrophils [28][29][30]. IC causes a marked decrease in the TNF-α concentration in hepatic homogenates after one hour of reperfusion. This effect persists during further phases of reperfusion (6 and 72 hrs), but the difference between the IC rats and the IR group is not statistically significant. The results obtained in our experimental study correspond to the results of Vajdova et al. obtained in mice after three hours of reperfusion [31]. Therefore, it can be assumed that a beneficial effect of IC reduces the activation of KC in the early phase of reperfusion. It has been suggested that KCs are also target cells for ischemic preconditioning, since it is not effective in the liver in which these cells have been blocked [32]. A similar phenomenon can also occur in IC of the portal triad. Reperfusion injury is closely related to an inflammatory response that attracts neutrophils to the organ where hepatocytes are damaged. The experiments involving the inhibition of neutrophil activity during IR, endotoxic shock, or blocking bile flow from the liver, have brought about long-lasting protection of the liver from injury [2][33][34]. Myeloperoxidase is the enzyme present in neutrophil granules, as well as in monocytes and macrophages but in smaller amounts. In normal conditions, MPO is not active in the liver and that is why it is a good indicator of neutrophil accumulation and activation. In our experimental study, IC of the portal triad reduced the MPO activity in the early phase of reperfusion (1 and 6 hrs) but did not affect the late phase of the injury (72 hrs). Taking into consideration a suggestion that IC protective mechanism may resemble ischemic preconditioning, similar results were obtained by Peralta et al. [35]. Among the mechanisms observed during reperfusion, damage of membranes due to lipid peroxidation is frequently mentioned. The evaluation of lipid peroxidation is an important and commonly used evaluation criterion of cell damage during IR of the liver. It is usually evaluated by the measurement of the main primary products of peroxidation, i.e., conjugated dienes and lipid hydroxides and/or their disintegration products (MDA, hexyl-cinnamic aldehyde, volatile carbohydrates) [36]. The results we obtained for MDA in the liver homogenates indicated that cytoplasmic membranes had been protected from ROS damage in all studied periods. Uchinami et al. confirmed that IC of the portal triad was an effective means of reducing the toxic activity of ROS, when they compared ROS formation in rats underwent either to continuous or intermittent portal triad clamping. These authors found that after 60-min. intermittent portal triad clamping, fewer ROS were formed than after continuous clamping, but their number was still relatively high [11]. Ozmen et al. showed that IC of the portal triad decreased the serum MDA concentration after 1 and 6 hrs of reperfusion when compared to 30-min continuous clamping [12]. We used an immunohistochemical method to evaluate the extent of hepatocyte apoptosis. The employed antibody binds to the site of the caspase cleavage of cytokeratin 18 during early phase of apoptosis, thereby facilitates the determination of single apoptotic hepatocytes [37][38]. IC of the portal triad employed in our experimental study protected hepatocytes from apoptosis caused by IR of the liver in early (1 hr) and late (72 hrs) phases of reperfusion. After six hours of reperfusion, the number of cells that underwent apoptosis was lower than that in IR rats, but the difference was not statistically significant. Our results were in accord to the findings published by others [6][7][8]. Crenesse et al. thought that IC could decrease the number of cell death by apoptosis through the reduction of ROS formation, or by inhibition of KC activity, which also decreases the release of TNF-α[8]. The obtained results may confirm both hypotheses, though they indicate that IC rather reduces the effects of oxidative stress (reducing ROS formation) than limits the release of TNF-α. In our study, the influence of IC on TNF-α formation was only evident after one hour of reperfusion. This may suggest that limiting the apoptosis of hepatocytes due to IC is related mostly to a decrease in oxidative stress. Nonetheless, inhibiting TNF-α release by the Browicz-Kupffer cells may have a significant effect on this limiting of apoptosis in the early phase of reperfusion. It should be added that Jang et al. showed that the use of IC during 60-min ischemia of a cirrhotic liver of mice reduced the number of cells that died of apoptosis by inhibiting the activation of the apoptotic pathway induced by inner mitochondria-related signalling [39]. Therefore, a detailed mechanism of apoptosis inhibition due to IC requires further studies. The obtained results indicate that IC protects hepatocytes from ischemia-reperfusion injury. In the early phase of reperfusion, IC reduces the release of TNF-α, which may be related to a beneficial effect of this method in further phases of reperfusion, including the reduction in neutrophil infiltration and number of apoptotic cells. It seems that the reduction of lipid peroxidation may limit the liver injury during reperfusion.
